# Establishment and validation of a prognosis nomogram for MIMIC-III patients with liver cirrhosis complicated with hepatic encephalopathy

**DOI:** 10.1186/s12876-023-02967-1

**Published:** 2023-09-28

**Authors:** Wansheng Yan, Zhihui Yao, Qiutong Ou, Gang Ye

**Affiliations:** 1 Department of Gastroenterology, The First Affiliated Hospital of Jinan University, Guangzhou, 510630 China; 2 Department of Colorectal Surgery, The Sixth Affiliated Hospital, Sun Yat-Sen University, Guangzhou, 510655 China

**Keywords:** MIMIC-III database, Hepatic encephalopathy, Cirrhosis, Prognosis, Nomogram, Sequential organ failure assessment score

## Abstract

**Introduce:**

The purpose of this study was to establish a comprehensive prognosis nomogram for patients with liver cirrhosis complicated with hepatic encephalopathy (HE) in the intensive care unit (ICU) and to evaluate the predictive value of the nomogram.

**Method:**

This study analyzed 620 patients with liver cirrhosis complicated with HE from the Medical Information Mart for Intensive Care III(MIMIC-III) database. The patients were randomly divided into two groups in a 7-to-3 ratio to form a training cohort (*n* = 434) and a validation cohort (*n* = 176). Cox regression analyses were used to identify associated risk variables. Based on the multivariate Cox regression model results, a nomogram was established using associated risk predictor variables to predict the 90-day survival rate of patients with cirrhosis complicated with HE. The new model was compared with the Sequential organ failure assessment (SOFA) scoring model in terms of the concordance index (C-index), the area under the curve (AUC) of receiver operating characteristic (ROC) analysis, the net reclassification improvement (NRI), the integrated discrimination improvement (IDI), calibration curve, and decision curve analysis (DCA).

**Results:**

This study showed that older age, higher mean heart rate, lower mean arterial pressure, lower mean temperature, higher SOFA score, higher RDW, and the use of albumin were risk factors for the prognosis of patients with liver cirrhosis complicated with HE. The use of proton pump inhibitors (PPI) was a protective factor. The performance of the nomogram was evaluated using the C-index, AUC, IDI value, NRI value, and DCA curve, showing that the nomogram was superior to that of the SOFA model alone. Calibration curve results showed that the nomogram had excellent calibration capability. The decision curve analysis confirmed the good clinical application ability of the nomogram.

**Conclusion:**

This study is the first study of the 90-day survival rate prediction of cirrhotic patients with HE in ICU through the data of the MIMIC-III database. It is confirmed that the eight-factor nomogram has good efficiency in predicting the 90-day survival rate of patients.

## Introduction

Hepatic encephalopathy (HE) is a severe brain dysfunction secondary to liver insufficiency or portal shunt, in which clinical symptoms vary greatly from slight mental disorder to coma [1]. Most patients with liver cirrhosis have different severity of HE during the development of the disease. According to reports, the incidence of overt HE in patients with liver cirrhosis is about 30%-45% [2], while the incidence of minimal HE is even higher, about 30%-85% [3–5]. Although HE is a comprehensive reversible disease, its low survival rate, high recurrence rate, and sudden changes in cognitive function burden the family and society of patients. When patients with liver cirrhosis develop into HE, they consume more medical resources, increase medical expenses, and prolong hospital duration. Grishma Hirode et al. found that from 2010 to 2014, the total number of hospitalizations for patients with HE in the United States increased by 24.4% (25,059 in 2010 and 31,182 in 2014, *p* < 0.001), and total hospitalization costs increased by 46.0% ($8.15 billion in 2010 and $11.9 billion in 2014, *P* < 0.001) [6]. Especially when patients with cirrhosis complicated with HE need to be admitted to the ICU for treatment, the more severe the patient’s condition and the higher the medical burden. Therefore, it is crucial to identify the risk factors of patients with liver cirrhosis complicated with HE in ICU and intervene in advance to prevent aggravation.

By far, there is no specific survival prediction model for patients with HE in the ICU. The severity scores of critically ill patients commonly used in ICU include the Sequential organ failure assessment (SOFA) score, the model for end-stage liver disease (MELD) score, and so on. The model for the MELD score was first proposed by Malinchoc et al. to predict the mortality of end-stage liver disease undergoing jugular intrahepatic portosystemic shunt [7]. It was found that the MELD score can be used as a predictor of the length of hospitalization in patients with HE [8]. SOFA score can be used to describe the severity of multiple organ failure by calculating scores through objective and easily available indicators. The SOFA score’s main content includes assessing six major organ systems: respiratory, cardiovascular, liver, kidney, nervous, and blood [9]. Currently, the SOFA score is widely used to predict the mortality of various critical diseases, such as sepsis, acute pancreatitis, etc. [10, 11]. The third international consensus definition of sepsis and septic shock (Sepsis-3) in 2016 shows that the change of SOFA score has become a vital component of the diagnosis criteria of sepsis [12].

Currently, prognostic systems based on risk scores have been widely used in critically ill patients [13]. However, using SOFA or MELD scores alone for predicting disease death still has limitations, which do not consider the influence of demographic factors or treatment measures.

This study aimed to determine the risk factors related to the 90-day survival of patients with liver cirrhosis and HE in ICU and to establish a new prognostic nomogram based on the results of the multivariate Cox regression. The new nomogram was compared with that of the separate SOFA model, and its performance was verified in the validation cohort.

## Materials and methods

### Data source

Data mining techniques are increasingly being used in big clinical data and public healthcare databases for the benefit of people [14]. This study mainly retrieves data from the Medical Information Mart for Intensive Care III database version 1.4(MIMIC-III v1.4). MIMIC-III database is an extensive, open, single-center intensive care database that collected health data of more than 50,000 patients hospitalized in Beth Israel Deaconess Medical Center from 2001 to 2012 [15]. To access the MIMIC-III database, the author completed the “Protection of Human Research Participants” course and obtained certification (researcher certificate number 36482492). The use of the MIMIC-III database was approved by the Institutional Review Boards of Beth Israel Deaconess Medical Center (Boston, MA) and the Massachusetts Institute of Technology (Cambridge, MA). All procedures performed in the present study were in accordance with the principles outlined in the 1964 Helsinki Declaration and its later amendments. MIMIC-III data is publicly available, and the personal privacy information of patients in this database is de-identified. So, this study was exempted from obtaining informed consent by the institutional research committee of the First Affiliated Hospital of Jinan University (Guangzhou, China).

### Patients and data extraction

**Fig. 1:**
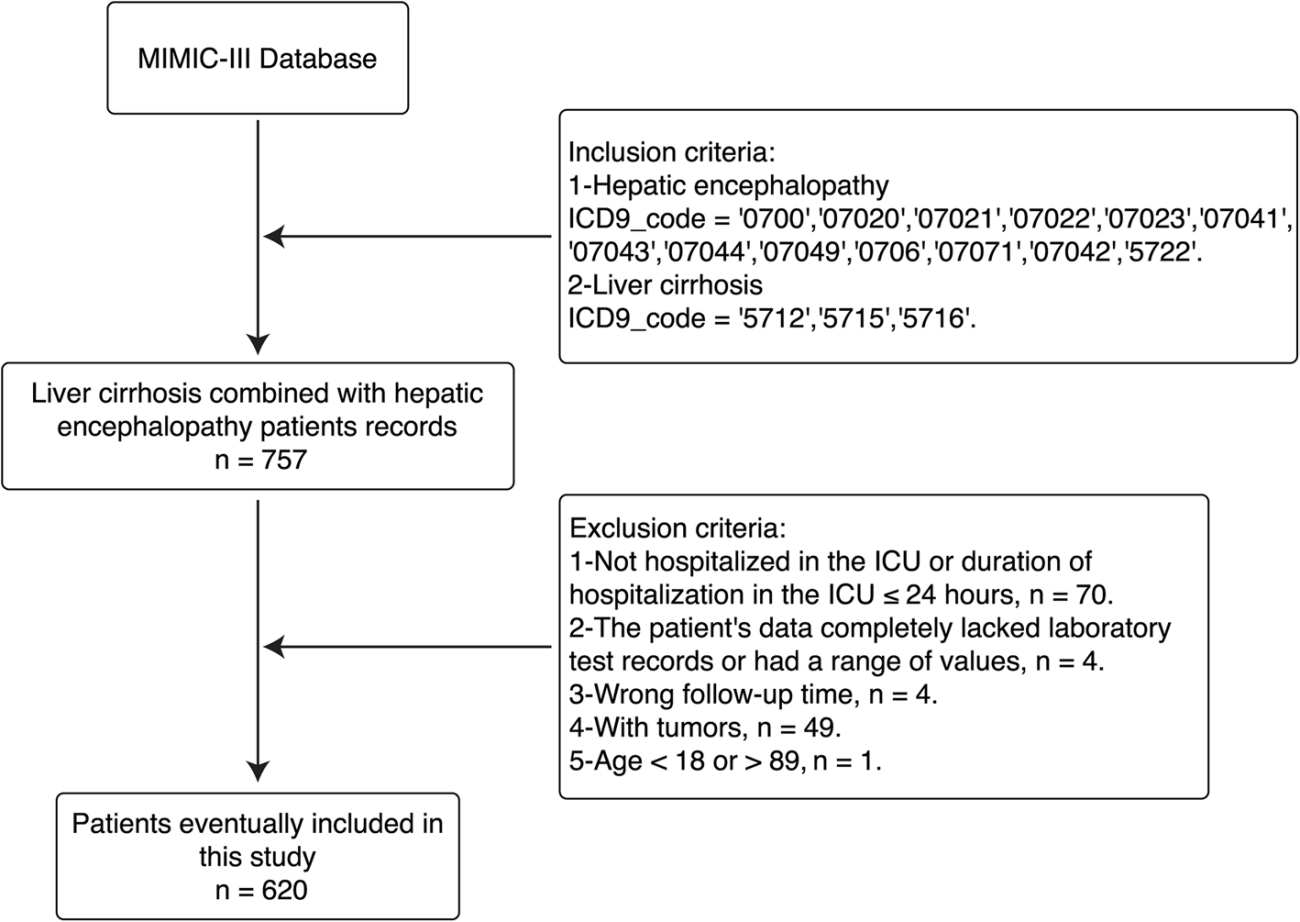
The screening process of the study sample. MIMIC-III, Medical Information Mart for Intensive Care III

Patients enrolled in this study were hospitalized in the ICU and diagnosed with cirrhosis complicated with HE at discharge. The exclusion criteria were as follows: (1) Not hospitalized in the ICU or duration of hospitalization in the ICU ≤ 24 h, (2) The patient’s data completely lacked laboratory test records or had a range of values, (3) Wrong follow-up time, (4) Patients with tumors, (5) Age < 18 or > 89. The screening process is shown in Fig. 1.

The relevant data of patients were extracted from the MIMIC-III database by executing the structured query language. According to the ninth edition of the International Classification of Diseases (ICD-9), the ICD-9 codes 0700,07020,07021,07022,07023,07041,07043,07044,07049,0706,07071,07042,5722, were used to extract information of patients diagnosed with HE (including hepatic coma). Then, ICD-9 codes 5712,5715,5716 were used to extract information about patients diagnosed with liver cirrhosis. If patients had records of multiple hospitalizations or admissions to the ICU, the first ICU records of each hospitalization were included in the study.

The data extracted from the MIMIC-III database in this study included demographic factors, average vital signs on the first day in the ICU, urine output in the first 24 h of ICU, first laboratory examination results after admission, comorbidities, SOFA score, and MELD score. In addition, information about therapeutic measures during hospitalization was also extracted. Ninety days survival after discharge was used as the endpoint of this study. The survival time was based on the time from discharge to death recorded by the Social Security Administration.

### Data pre-processing

In this study, variables with missing data of more than 20% were excluded. For the variables with less than 20% missing data, the multiple imputation method was used to fill in missing values with predictor variables by the “MICE” package of R software [16]. Finally, 47 variables included in the study were as follows:(1) basic Information, including age, gender, weight, and etiology of liver cirrhosis; (2) mean vital signs on the first day of ICU admission,including mean heart rate, mean arterial pressure(MAP), mean temperature, mean blood oxygen saturation(SpO2), mean respiratory rate and 24 h urine output; (3) comorbidities, including alcohol abuse, diabetes, hypertension, cardiac arrhythmias, congestive heart failure, coagulopathy, chronic pulmonary and renal failure; (4) first laboratory examination results after admission, including lactate, albumin, serum alkaline phosphatase(ALP), alanine aminotransferase(ALT), aspartate aminotransferase(AST), anion gap, bicarbonate, chloride, magnesium, potassium, sodium, total bilirubin, total calcium, urea nitrogen, creatinine, hemoglobin, international normalized ratio(INR), platelets (PLT), prothrombin time (PT), partial thromboplastin time (PTT), red blood cell count (RBC), red blood cell distribution width (RDW), white blood cell count (WBC); (5)disease severity scores, including SOFA score and MELD Score; (6) therapeutic measures during hospitalization, including the use of albumin, proton pump inhibitors(PPI), furosemide and percutaneous abdominal drainage (PAD).

The data set was randomly divided into training and validation cohorts at 7: 3 ratios. The training cohort was used to establish the nomogram, and the validation cohort to verify it.

### Statistical analysis

Statistical analysis of the baseline data was performed using IBM SPSS statistical software (version 21.0, IBM Corp). The Shapiro–Wilk test was first applied to determine the distribution of continuous variable data. Continuous variables were expressed as mean ± SD or median (IQR), and differences between two groups were assessed by t-test or rank sum test. Categorical variables were expressed as frequency (percentage), and differences between the two groups were evaluated by chi-square test. Statistical significance was defined as *P* < 0.05.

Univariate Cox regression analysis was applied to all variables. The variables with *P* < 0.1 in the univariate Cox regression results were included in the multivariate Cox regression analysis. According to the results of multivariate analysis, variables with *P* value < 0.05 or specific clinical application significance were included in the final model. The Cox zph function of the survival package in R software was used to determine whether the new model met the requirements of the proportional hazard. The new model would be presented in the form of a nomogram.

The prediction accuracy of the nomogram was evaluated by the C-index and the area under the curve (AUC) of receiver operating characteristic (ROC) analysis [17]. Then, net reclassification improvement (NRI) [18] and integrated discrimination improvement (IDI) [19] were applied to assess the overall improvement in the predictive power of the new nomogram compared to the SOFA scoring model alone. The calibration curve was applied to evaluate the calibration ability of the nomogram [20]. In addition, decision curve analysis (DCA) [21] was used to assess the net clinical benefit of the nomogram. R software (version 4.0.3) mainly carried out the above analysis.

## Results

A total of 620 patients were enrolled in the study. According to the 7:3 random allocation, the training and validation cohorts consisted of 434 and 186 patients, respectively. All baseline characteristics of the training and validation cohorts are shown in Table 1. The median age of patients was 54.72 years in the training cohort and 54.79 years in the validation cohort. Most patients in the training and validation cohorts were male (63.8% and 65.6%, respectively). The 90-day survival rate for the training cohort was 53.69%, and the 90-day survival rate for the validation cohort was 56.45%. Baseline information on survivors and deceased patients in the training and validation cohorts are shown in Tables 2 and 3, respectively. Table 2 shows the factors that showed significant differences between groups of survivors and deaths in the training cohort, including (*p* < 0.05): age, MAP, mean respiratory rate, mean SpO2, mean temperature, cardiac arrhythmias, lactate, albumin, anion gap, total bilirubin, chloride, creatinine, magnesium, potassium, sodium, urea nitrogen, INR, PT, PTT, RDW, WBC, albumin use, furosemide use, PAD, SOFA, MELD, and urine output. Table 3 shows the factors that showed significant differences between groups of survivors and deaths in the validation cohort, including (*p* < 0.05): MAP, mean SpO2, mean temperature, cardiac arrhythmias, congestive heart failure, ALT, albumin, AST, total bilirubin, creatinine, magnesium, potassium, sodium, urea nitrogen, INR, PT, PTT, RDW, WBC, albumin use, PAD, SOFA, MELD, and urine output.

**Table 1:** Characteristics at baseline of patients in the study

**Variable**	**Training cohort (*n* = 434)**	**Validation cohort (*n* = 186)**	***P* value**
**Demographics**			
Age, year	54.72 (14.91)	54.79 (11.96)	0.496
Gender, n(%)			0.674
Men	277 (63.8)	122 (65.6)	
Women	157 (36.2)	64 (34.4)	
weight, kg	85.00 (26.00)	82.00 (29.00)	0.899
**Etiology of cirrhosis**			0.689
Alcoholic cirrhosis of liver	257 (59.2%)	111 (59.7%)	
Cirrhosis of liver without mention of alcohol	168 (38.7%)	73 (39.2%)	
Biliary cirrhosis	9 (2.1%)	2 (1.1%)	
**Firstday vital signs in ICU**^a^			
Mean heart rate, beats/min	87.09 (23.13)	83.67 (22.65)	0.021
MAP, mmHg	73.75 (15.38)	72.82 (17.35)	0.255
Mean respiratory rate, breaths/min	17.87 (5.32)	17.52 (4.99)	0.918
Mean SpO2,%	97.64 (2.78)	97.74 (2.43)	0.721
Mean temperature, °C	36.60 (0.83)	36.57 (0.83)	0.859
**Complication**			
Alcohol abuse, n(%)	256 (59.0)	107 (57.5)	0.735
Chronic pulmonary, n(%)	78 (18.0)	38 (20.4)	0.472
Cardiac arrhythmias, n(%)	79 (18.2)	32 (17.2)	0.766
Coagulopathy, n(%)	245 (56.5)	103 (55.4)	0.805
Congestive heart failure, n(%)	68 (15.7)	29 (15.6)	0.981
Diabetes, n(%)	123 (28.3)	56 (30.1)	0.656
Hypertension, n(%)	149 (34.3)	65 (34.9)	0.883
Renal failure, n(%)	83 (19.1)	44 (23.7)	0.200
**First laboratory tests**^b^			
Lactate, mmol/L	2.30 (1.80)	2.20 (1.40)	0.191
ALT,IU/L	37.00 (39.00)	37.50 (44.00)	0.285
Albumin, g/dL	2.80 (0.70)	2.80 (0.80)	0.536
ALP, IU/L	125.00 (87.00)	130.50 (96.00)	0.277
Anion gap, mEq/L	14.00 (6.00)	15.00 (6.00)	0.594
AST,IU/L	72.00 (81.00)	72.00 (92.00)	0.790
Bicarbonate, mEq/L	22.00 (7.00)	21.00 (7.00)	0.324
Total bilirubin, mg/dL	4.20 (6.70)	4.35 (8.90)	0.910
Total calcium, mg/dL	8.40 (1.10)	8.40 (1.00)	0.628
Chloride, mEq/L	103.00 (11.00)	101.00 (10.00)	0.451
Creatinine, mg/dL	1.30 (1.40)	1.45 (1.70)	0.344
Magnesium, mg/dL	1.90 (0.50)	2.00 (0.60)	0.155
Potassium, mEq/L	4.30 (1.20)	4.35 (1.20)	0.057
Sodium, mEq/L	136.00 (9.00)	134.50 (9.00)	0.169
Urea Nitrogen, mg/dL	30.00 (32.00)	31.00 (34.00)	0.140
Hemoglobin, g/dL	10.40 (2.60)	10.25 (2.30)	0.594
INR	1.80 (0.80)	1.80 (0.70)	0.891
PLT,K/uL	101.50 (80.00)	97.50 (75.00)	0.831
PT, sec	18.60 (6.20)	18.10 (5.90)	0.746
PTT, sec	38.80 (12.60)	39.90 (12.60)	0.530
RDW,%	17.40 (3.10)	17.00 (3.30)	0.580
WBC, K/uL	8.50 (6.70)	8.20 (6.80)	0.791
RBC, m/uL	3.16 (0.82)	3.11 (0.76)	0.643
**Therapeutic measure**			
Albumin use, n(%)	211 (48.6%)	92 (49.5%)	0.847
Furosemide use, n(%)	232 (53.5%)	93 (50.0%)	0.430
PPI use, n(%)	331 (76.3%)	142 (76.3%)	0.984
PAD, n(%)	226 (52.1%)	89 (47.8%)	0.335
**Severity score**			
SOFA	9.00 (5.00)	9.00 (5.00)	0.959
MELD	30.00 (12.64)	30.00 (13.96)	0.556
**Others**			
Urine Output^c^, ml	964.50 (1044.00)	1029.00 (1161.00)	0.878

Categorical variables were presented as frequency (percentage), and continuous variables were presented as median (interquartile)

*MAP* Mean arterial pressure, *SpO2* Blood oxygen saturation, *ALT* Alanine aminotransferase, *AST* Aspartate aminotransferase, *ALP* Alkaline phosphatase, *INR* International normalized ratio, *PT* Prothrombin time, *PTT* Partial thromboplastin time, *PLT* Platelet, *RDW* Red cell distribution width, *WBC* White blood cell count, *RBC* Red blood cell count, *PPI.use* Proton pump inhibitors use, *PAD* Percutaneous abdominal drainage, *SOFA* Sequential organ failure assessment, *MELD* Model for end-stage liver disease

^a^Vital signs were calculated as the mean value during the first 24 h since ICU admission of each included patients

^b^The laboratory tests recorded the first value after admission

^c^The urine output was recorded during the first 24 h in the ICU

**Table 2:** Characteristics at baseline of patients in the training cohort

**Variable**	**Alive (*n* = 233)**	**Dead (*n* = 201)**	***P* value**
**Demographics**			
Age, year	53.51 (14.89)	56.40 (15.65)	0.016
Gender, n(%)			0.674
Men	143 (61.4)	134 (66.7)	
Women	90 (38.6)	67 (33.3)	
weight, kg	85.00 (25.00)	84.00 (26.00)	0.585
**Etiology of cirrhosis(%)**			0.896
Alcoholic cirrhosis of liver	139 (59.7)	118 (58.7)	
Cirrhosis of liver without mention of alcohol	90 (38.6)	78 (38.8)	
Biliary cirrhosis	4 (1.7)	5 (2.5)	
**Firstday vital signs in ICU**^a^			
Mean heart rate, beats/min	86.57 (22.44)	88.08 (24.61)	0.081
MAP, mmHg	77.06 (15.90)	71.31 (11.99)	< 0.001
Mean respiratory rate, breaths/min	17.23 (5.21)	18.60 (5.18)	0.016
Mean SpO2,%	98.00 (2.64)	97.40 (2.80)	0.002
Mean temperature, °C	36.72 (0.80)	36.42 (0.76)	< 0.001
**Complication**			
Alcohol abuse, n(%)	143 (61.4)	113 (56.2)	0.276
Chronic pulmonary, n(%)	45 (19.3)	33 (16.4)	0.433
Cardiac arrhythmias, n(%)	34 (14.6)	45 (22.4)	0.036
Coagulopathy, n(%)	126 (54.1)	119 (59.2)	0.283
Congestive heart failure, n(%)	36 (15.5)	32 (15.9)	0.893
Diabetes, n(%)	59 (25.3)	64 (31.8)	0.133
Hypertension, n(%)	78 (33.5)	71 (35.3)	0.686
Renal failure, n(%)	41 (17.6)	42 (20.9)	0.384
**First laboratory tests**^b^			
Lactate, mmol/L	2.20 (1.30)	2.70 (2.15)	0.001
ALT, IU/L	36.00 (32.00)	40.00 (48.00)	0.207
Albumin, g/dL	2.80 (0.70)	2.70 (0.70)	0.041
ALP, IU/L	123.00 (80.00)	128.00 (102.00)	0.272
Anion gap, mEq/L	14.00 (6.00)	15.00 (6.00)	0.023
AST, IU/L	69.00 (80.00)	75.00 (86.00)	0.168
Bicarbonate, mEq/L	22.00 (7.00)	22.00 (7.00)	0.685
Total bilirubin, mg/dL	3.60 (4.60)	5.50 (10.90)	< 0.001
Total calcium, mg/dL	8.30 (1.10)	8.40 (1.20)	0.183
Chloride, mEq/L	103.00 (10.00)	101.00 (12.00)	0.002
Creatinine, mg/dL	1.20 (1.40)	1.50 (1.50)	0.004
Magnesium, mg/dL	1.90 (0.50)	2.00 (0.60)	0.014
Potassium, mEq/L	4.10 (1.10)	4.40 (1.20)	0.021
Sodium, mEq/L	137.00 (8.00)	134.00 (10.00)	0.002
Urea Nitrogen, mg/dL	24.00 (30.00)	33.00 (30.00)	< 0.001
Hemoglobin, g/dL	10.20 (2.60)	10.40 (2.70)	0.641
INR	1.70 (0.80)	1.90 (0.70)	0.005
PLT, K/uL	100.00 (88.00)	102.00 (75.00)	0.750
PT, sec	18.10 (5.40)	19.20 (6.50)	0.007
PTT, sec	37.80 (11.60)	40.50 (14.20)	0.006
RDW,%	17.10 (3.20)	17.90 (3.20)	0.002
WBC, K/uL	8.00 (6.70)	9.10 (7.80)	0.015
RBC, m/uL	3.13 (0.86)	3.17 (0.82)	0.639
**Therapeutic measure**			
Albumin use, n(%)	89 (38.2%)	122 (60.7%)	< 0.001
Furosemide use, n(%)	138 (59.2%)	94 (46.8%)	0.009
PPI use, n(%)	185 (79.4%)	146 (72.6%)	0.099
PAD, n(%)	104 (44.6%)	122 (60.7%)	0.001
**Severity score**			
SOFA	8.00 (4.00)	9.00 (5.00)	< 0.001
MELD	22.64 (11.65)	30.33 (11.32)	< 0.001
**Others**			
Urine Output^c^, ml	964.50 (1044.00)	1029.00 (1161.00)	< 0.001

Categorical variables were presented as frequency (percentage), parametric continuous variables were presented as median (interquartile)

*MAP* Mean arterial pressure, *SpO2* Blood oxygen saturation, *ALT* Alanine aminotransferase, *AST* Aspartate aminotransferase, *ALP* Alkaline phosphatase, *INR* International normalized ratio, *PT *Prothrombin time, *PTT* Partial thromboplastin time, *PLT* Platelet, *RDW* Red cell distribution width, *WBC* White blood cell count, *RBC* Red blood cell count, *PPI.use* Proton pump inhibitors use, *PAD* Percutaneous abdominal drainage, *SOFA* Sequential organ failure assessment, *MELD* Model for end-stage liver disease

^a^Vital signs were calculated as mean value during the first 24 h since ICU admission of each included patients

^b^The laboratory tests recorded the first value after admission

^c^The urine output was recorded during the first 24 h in the ICU

**Table 3:** Characteristics at baseline of patients in the validation cohort

**Variable**	**Alive (*n* = 105)**	**Dead (*n* = 81)**	***P* value**
**Demographics**			
Age, year	54.49 (12.51)	56.87 (12.34)	0.364
Gender, n(%)			0.330
Men	72 (68.6)	50 (61.7)	
Women	33 (31.4)	31 (38.3)	
weight, kg	84.00 (28.00)	80.00 (26.00)	0.303
**Etiology of cirrhosis(%)**			0.230
Alcoholic cirrhosis of liver	66 (62.9)	45 (55.6)	
Cirrhosis of liver without mention of alcohol	37 (35.2)	36 (44.4)	
Biliary cirrhosis	2 (1.9)	0 (0.0)	
**Firstday vital signs in ICU**^a^			
Mean heart rate, beats/min	82.54 (22.83)	84.00 (23.44)	0.335
MAP, mmHg	74.84 (15.32)	68.67 (11.88)	0.001
Mean respiratory rate, breaths/min	17.37 (4.48)	18.64 (5.73)	0.069
Mean SpO2,%	98.15 (2.41)	97.39 (2.65)	0.002
Mean temperature, °C	36.71 (0.78)	36.35 (0.84)	0.002
**Complication**			
Alcohol abuse, n(%)	60 (57.1)	47 (58.0)	0.904
Chronic pulmonary, n(%)	17 (16.2)	21 (25.9)	0.103
Cardiac arrhythmias, n(%)	12 (11.4)	20 (24.7)	0.017
Coagulopathy, n(%)	53 (50.5)	50 (61.7)	0.126
Congestive heart failure, n(%)	10 (9.5)	19 (23.5)	0.009
Diabetes, n(%)	28 (26.7)	28 (34.6)	0.244
Hypertension, n(%)	32 (30.5)	33 (40.7)	0.145
Renal failure, n(%)	21 (20.0)	23 (28.4)	0.182
**First laboratory tests**^b^			
Lactate, mmol/L	2.20 (1.25)	2.30 (1.90)	0.258
ALT,IU/L	34.00 (35.00)	47.00 (73.00)	0.039
Albumin, g/dL	2.90 (0.80)	2.70 (0.80)	0.004
ALP,IU/L	127.00 (94.00)	137.00 (94.00)	0.111
Anion gap, mEq/L	14.00 (5.00)	15.00 (6.00)	0.436
AST,IU/L	61.00 (55.00)	96.00 (122.00)	0.016
Bicarbonate, mEq/L	22.00 (6.00)	21.00 (8.00)	0.314
Total bilirubin, mg/dL	3.50 (4.70)	6.10 (15.3)	< 0.001
Total calcium, mg/dL	8.50 (1.00)	8.20 (1.10)	0.172
Chloride, mEq/L	102.00 (11.00)	100.00 (11.00)	0.055
Creatinine, mg/dL	1.20 (1.40)	1.60 (2.00)	0.013
Magnesium, mg/dL	2.00 (0.60)	2.10 (0.60)	0.030
Potassium, mEq/L	4.20 (1.20)	4.70 (1.20)	0.003
Sodium, mEq/L	135.00 (9.00)	134.00 (10.00)	0.015
Urea Nitrogen, mg/dL	28.00 (32.00)	36.00 (32.00)	0.005
Hemoglobin, g/dL	10.20 (2.20)	10.30 (2.40)	0.728
INR	1.70 (0.50)	2.00 (1.10)	< 0.001
PLT,K/uL	98.00 (75.00)	96.00 (80.00)	0.911
PT, sec	16.70 (4.10)	21.00 (9.60)	< 0.001
PTT, sec	36.50 (12.10)	43.90 (13.20)	< 0.001
RDW,%	16.70 (3.30)	17.80 (4.00)	0.029
WBC,K/uL	7.20 (4.8)	9.40 (8.50)	0.001
RBC, m/uL	3.07 (0.84)	3.13 (0.71)	0.958
**Therapeutic measure**			
Albumin use, n(%)	45 (42.9%)	47 (58.0%)	0.040
Furosemide use, n(%)	51 (48.6%)	42 (51.9%)	0.657
PPI use, n(%)	84 (80.0%)	58 (71.6%)	0.182
PAD, n(%)	41 (39.0%)	48 (59.3%)	0.006
**Severity score**			
SOFA	8.00 (4.00)	10.00 (5.00)	0.001
MELD	21.32 (10.99)	30.99 (12.41)	< 0.001
**Others**			
Urine Output^c^, ml	1095.00 (1447.00)	816.00 (1186.00)	0.003

Categorical variables were presented as frequency (percentage), parametric continuous variables were presented as median (interquartile)

*MAP* Mean arterial pressure, *SpO2* Blood oxygen saturation, *ALT* Alanine aminotransferase, *AST* Aspartate aminotransferase, *ALP* Alkaline phosphatase, *INR* International normalized ratio, *PT *Prothrombin time, *PTT *Partial thromboplastin time, *PLT* Platelet, *RDW* Red cell distribution width, *WBC* White blood cells count, *RBC* Red blood cell count, *PPI.use* Proton pump inhibitors use, *PAD* Percutaneous abdominal drainage, *SOFA* Sequential organ failure assessment, *MELD* Model for end-stage liver disease

^a^Vital signs were calculated as mean value during the first 24 h since ICU admission of each included patients

^b^The laboratory tests recorded the first value after admission

^c^The urine output was recorded during the first 24 h in the ICU

Univariate Cox regression analysis was performed on all baseline data factors initially included in the training cohort, and the results showed 28 potential predictors for 90-day survival, just as age, mean heart rate, MAP, mean temperature, mean SpO2, mean respiratory rate, cardiac arrhythmias, SOFA、MELD, lactate, urine output, albumin, total bilirubin, urea nitrogen, sodium, potassium, magnesium, chloride, INR, RDW, WBC, ALP, PT, PTT, albumin use, PPI, PAD and furosemide. These candidate factors were input into a multivariate Cox regression analysis, and eight risk factors were found, including age (hazard ratio [HR] = 1.022, 95%Confidence interval [CI] = 1.006–1.037, *P* = 0.006), mean heart rate (HR = 1.013, 95%CI = 1.003–1.023, *P* = 0.010), SOFA (HR = 1.057, 95%CI = 0.998–1.119, *P* = 0.059), RDW (HR = 1.056, 95%CI = 0.994–1.122, *P* = 0.078), albumin use (HR = 1.428, 95%CI = 1.013–2.011, *P* = 0.042), MAP (HR = 0.982, 95%CI = 0.967–0.998, *P* = 0.031), mean temperature (HR = 0.731, 95%CI = 0.554–0.996, *P* = 0.027) and PPI use (HR = 0.702, 95%CI = 0.500–0.985, *P* = 0.041). The results of the Cox regression analysis are shown in Table 4. The SOFA score and RDW were considered clinically significant for the prognosis of patients with cirrhosis and HE based on previous literature reports [22, 23] and clinical experience, so they were also included in the final prediction model.

**Table 4:** The results of Cox regression analysis

	Univariate cox model			Multivariable cox model		
Variables	HR	95%CI	*P* value	HR	95%CI	*P* value
Age	1.013	1.000–1.025	0.053	1.022	1.006–1.037	0.006
Mean SpO2	0.946	0.910–0.982	0.004			
Mean heart rate	1.008	1.000–1.016	0.044	1.013	1.003–1.023	0.010
MAP	0.965	0.952–0.978	< 0.001	0.982	0.967–0.998	0.031
Mean respiratory rate	1.038	1.008–1.070	0.014			
Mean temperature	0.583	0.465–0.731	< 0.001	0.731	0.554–0.966	0.027
Cardiac arrhythmias						
No	Reference					
Yes	1.468	1.054–2.046	0.023			
SOFA	1.106	1.064–1.150	< 0.001	1.057	0.998–1.119	0.059
MELD	1.037	1.022–1.051	< 0.001			
Lactate	1.090	1.033–1.141	0.001			
UrineOutput	1.000	1.000–1.000	< 0.001			
Albumin	0.776	0.603–0.999	0.049			
Total bilirubin	1.022	1.010–1.035	< 0.001			
Urea nitrogen	1.007	1.002–1.011	0.005			
Sodium	0.972	0.954–0.990	0.003			
Magnesium	1.491	1.132–1.964	0.004			
Chloride	0.969	0.953–0.986	< 0.001			
Potassium	1.159	1.016–1.323	0.028			
INR	1.175	1.029–1.342	0.018			
RDW	1.100	1.039–1.163	< 0.001	1.056	0.994–1.122	0.078
WBC	1.031	1.013–1.050	< 0.001			
ALP	1.001	1.000–1.002	0.058			
PT	1.018	1.000–1.036	0.048			
PTT	1.007	1.000–1.014	0.041			
PPI use	0.761	0.558–1.038	0.085	0.702	0.500–0.985	0.041
Furosemide use	0.698	0.529–0.921	0.011			
Albumin use	2.004	1.509–2.662	< 0.001	1.428	1.013–2.011	0.042
PAD	1.652	1.244–2.193	< 0.001			

*HR* hazard ratio, *CI* confidence interval, *MAP* Mean arterial pressure, *ALP* Alkaline phosphatase, *INR* International normalized ratio, *PT* Prothrombin time, *PTT* Partial thromboplastin time, *RDW* Red cell distribution width, *WBC* White blood cell count, *PPI.use* Proton pump inhibitors use, *PAD* Percutaneous abdominal drainage, *SOFA* Sequential organ failure assessment, *MELD* Model for end-stage liver disease

**Fig. 2:**
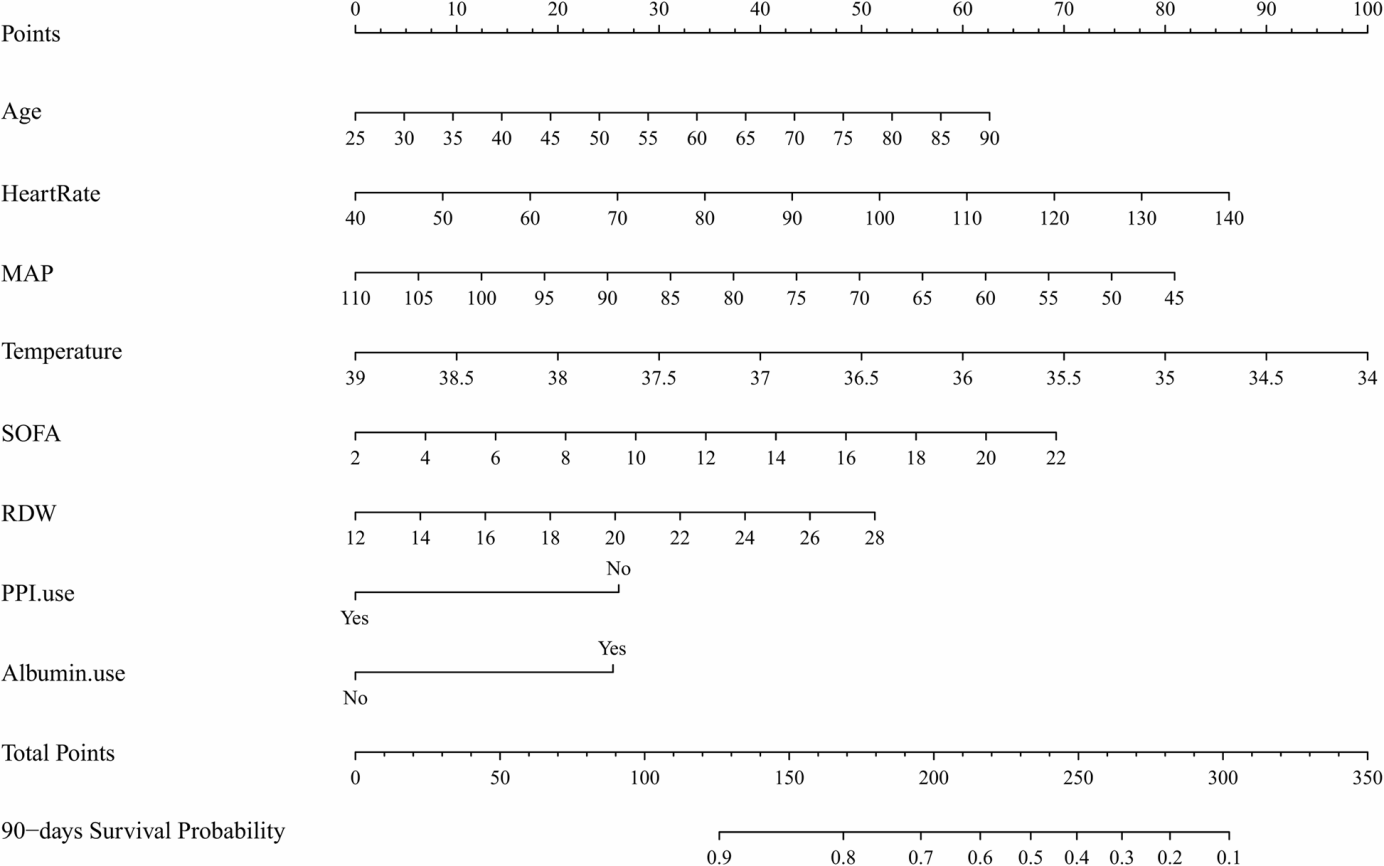
Nomogram for predicting the 90-day probability of survival from liver cirrhosis with hepatic encephalopathy. MAP, Mean arterial pressure; SOFA, Sequential organ failure assessment; RDW, Red cell distribution width; PPI.use, Proton pump inhibitors use

Based on the multivariate Cox regression analysis results, a nomogram about the 90-day survival rate of patients with liver cirrhosis and HE was constructed, as shown in Fig. 2. The nomogram indicated that age, higher SOFA score, higher RDW, higher mean heart rate, lower MAP, lower mean temperature, and the use of albumin were risk factors for the prognosis of patients, and the use of PPI was a protective factor.

**Fig. 3:**
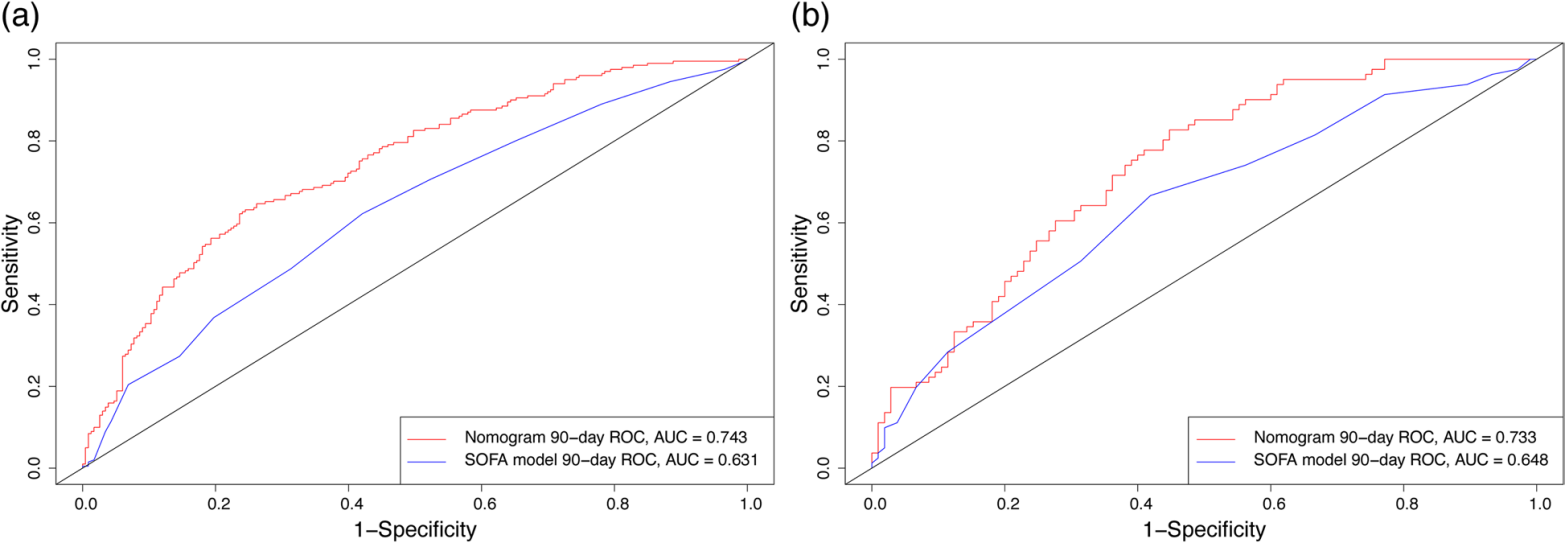
ROC curves for the nomogram and the SOFA mode. （**a**）: Result of the training cohort; （**b**）: Result of the validation cohort

The new nomogram was tested on the proportional hazard hypothesis, and the results showed that the *P* values of each factor and the overall *P* value were greater than 0.05, which conformed to the proportional hazard requirement. Then, C-index was used to evaluate the effect of the nomogram, which found that this was higher for the nomogram than for the single SOFA model in both the training cohort (0.704 versus 0.615) and the validation cohort (0.695 versus 0.638). In addition, the AUC value of the new nomogram was greater than that of the single SOFA model, both in the training cohort and the validation cohort. The ROC results are shown in Fig. 3.

The NRI value for the 90-day nomogram was 0.560(95%CI = 0.447–0.792) in the training cohort and 0.364 (95% CI = 0.054–0.756) in the validation cohort. In addition, the 90-day IDI value was 0.119 (*P* < 0.001) for the training cohort and 0.083 (*P* < 0.001)for the validation cohort, respectively. The NRI and IDI values obtained in this study were greater than zero, which indicated that the overall performance of the nomogram was better than that of the SOFA model alone.

**Fig. 4:**
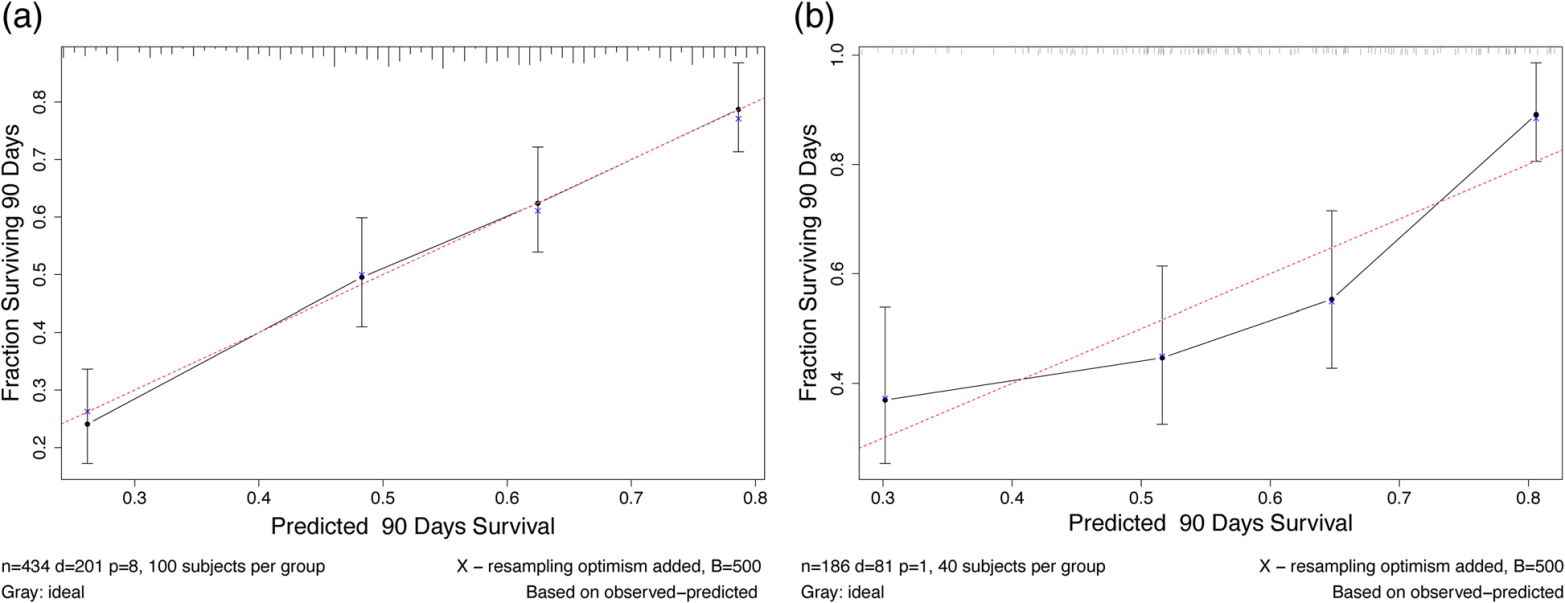
Calibration curves. Calibration curves for the 90-day probability of survival from liver cirrhosis with hepatic encephalopathy depict calibration of nomogram in terms of the agreement between the predicted probabilities and observed outcomes of the training cohort (**a**) and validation cohort (**b**)

Figure 4 shows the calibration curves of the training and validation cohort for the nomogram. The standard curve of the 90-day forecast probability of the nomogram was very close to the standard 45-degree diagonal line, and the relevant four tangent points were evenly distributed. The result showed that the new nomogram had excellent calibration capabilities.

**Fig. 5:**
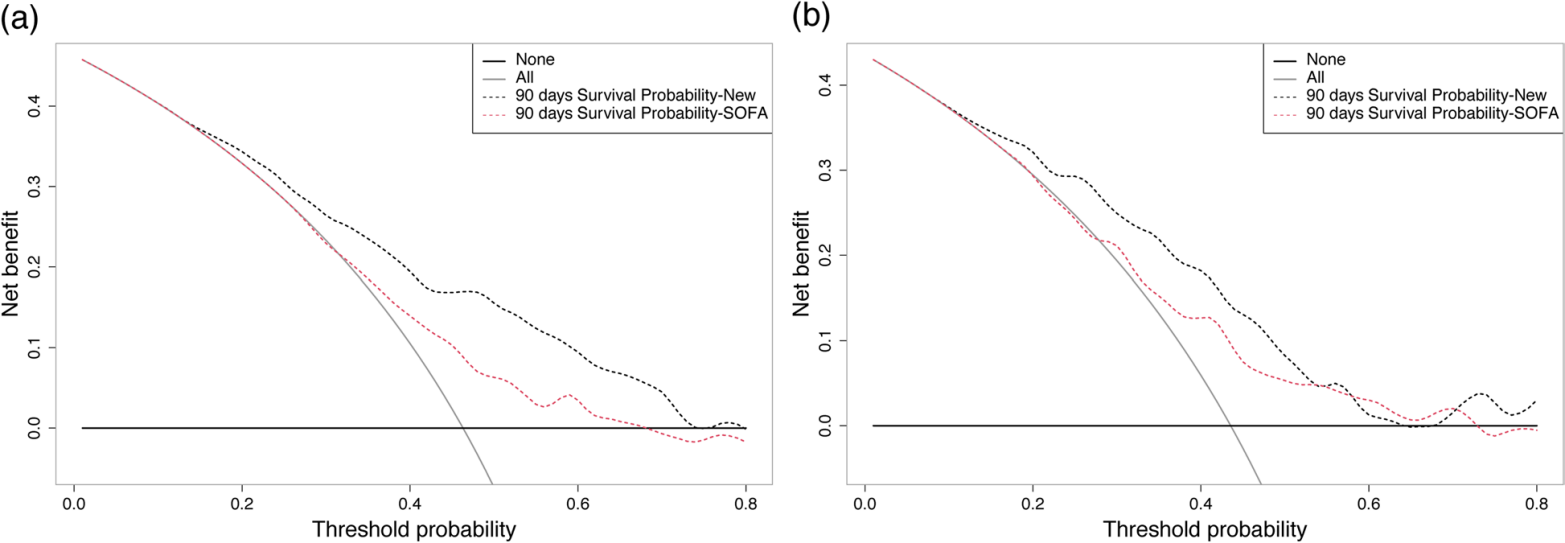
Decision curve for the new nomogram for 90-day prediction of survival probability in the training cohort (**a**) and validation cohort (**b**)

The DCA curves of the nomogram and the single SOFA model are shown in Fig. 5. The results demonstrated that the 90-day DCA curve of the nomogram produced a net benefit regardless of whether it was in the training cohort or the validation cohort, and the DCA curves of the nomogram were all enhanced, compared with the single SOFA model.

## Discussion

This study is the first to use the MIMIC-III database to study the 90-day survival prediction of patients with liver cirrhosis and HE in the ICU. At present, there is still a lack of a good prognosis prediction model in patients with liver cirrhosis and HE. Although many current disease severity scores, such as MELD and SOFA scores, have specific predictive power for the prognosis of patients [13], there is still a lack of consideration of some critical factors, such as RDW, the use of albumin, the use of proton pump inhibitors, etc.

This study focuses on vital signs, related laboratory indicators, disease severity scores, and the therapeutic measures of patients with liver cirrhosis and HE during hospitalization.

In this study, advanced age was an independent risk factor for poor prognosis in patients with liver cirrhosis combined with HE. The older the age, the worse the patient’s prognosis. This may be associated with decreased immune function and liver metabolic function, and changes in the gut-brain axis in older people [24]. In addition, it has been found that mild HE predisposes to falls [25], and older people are a vulnerable population, so those who develop HE are at higher risk for fall accidents.

The ICU physician pays close attention to the patient’s vital signs. The vital signs change is a significant indicator for physicians to directly judge the patient’s physical state and make the subsequent treatment decisions. Mean heart rate and mean arterial pressure(MAP) are the most common indicators of patient resuscitation that ICU physicians pay attention to. Mean heart rate and MAP are often used to reflect the patient’s cardiac function and blood volume. This study found that the higher the mean heart rate and the lower the MAP within 24 hours of admission to the ICU, the worse the prognosis of patients. The increased heart rate and decreased arterial pressure may reflect high dynamic circulation due to vascular dilation in the body’s viscera. Visceral vasodilation leads to hyperdynamic circulation syndrome, characterized by increased cardiac output and heart rate, decreased systemic vascular resistance, and low arterial blood pressure [26]. In cirrhosis, the dilation of visceral blood vessels can lead to increased visceral blood flow and the aggravation of portal hypertension, which can easily lead to HE [26]. Stable hemodynamics are critical to patient prognosis. Some scholars suggest that the MAP of patients with cirrhosis admitted to the ICU should be maintained above 65mmHg [27]. This study showed that the lower the average body temperature, the higher the mortality of patients. Abnormal body temperature is a common manifestation of critically ill patients in the ICU. Laupland KB et al. completed a study on the occurrence and determinants of abnormal body temperature within 24 h of visits to the ICU of 10,962 adult patients admitted to the French ICU from April 2000 to November 2010 and found that hypothermia is a significant independent predictor of death in medical patients [28]. Another study found that patients with hypothermia have worse clinical conditions and a worse prognosis [29]. These are consistent with the results of this research. SOFA score was a risk factor for the patients. The nomogram total score increased with the SOFA score. The prognosis of cirrhosis combined with HE is closely related to the number and degree of organ failure and the presence of infection. The SOFA score is generally used for the evaluation of multiple organ failure. The SOFA score is becoming a popular and essential tool for assessing the severity of disease or prognosis in critically ill patients [23]. Based on the SOFA score, many researchers have continued to explore and develop many scoring tools that can assess the severity of specific diseases, such as q-SOFA and time-incorporated SOFA [12, 30].

Red blood cell distribution width (RDW), as a simple and readily available biological index, has been paid much attention. RDW has been shown to be strongly associated with all-cause mortality and risk of bloodstream infection in critically ill patients, and it may reflect the overall inflammation, oxidative stress, or insufficient arterial filling of the patients [31]. RDW can be used as a potential prognostic indicator of liver disease [32], which is of great value in evaluating the severity of patients with acute decompensated liver cirrhosis [22] and patients with hepatitis B virus-related decompensated cirrhosis [33]. This study found that RDW was positively correlated with 90-day mortality in patients.

At present, the development of prognostic models related to cirrhosis combined with HE rarely incorporates therapeutic measures as research factors. In this study, therapeutic measures during hospitalization, such as the use of albumin and PPI, etc., were included, and the results showed that albumin infusion and PPI use were associated with the prognosis of the patients. Albumin plays a very powerful role in the human body. It can expand blood volume, improve microcirculation, bind and transport a variety of substances, and have excellent antioxidant properties [34, 35]. According to the comprehensive guidelines proposed by the American Association for the Study of Liver Diseases in 2021, the main indications for the use of albumin solutions in patients with cirrhosis are large-volume puncture, acute kidney injury, hepatorenal syndrome, and spontaneous bacterial peritonitis [36]. The efficacy of albumin infusion in patients with HE is still controversial. One study showed that albumin administration improved mortality in patients with cirrhosis and HE [37]. A Meta-analysis of human albumin infusion for cirrhosis and its complications found that in cirrhosis patients with overt HE, albumin infusion improved the severity of overt HE but not overall mortality [38]. In a randomized, double-blind, placebo-controlled trial about the effect of albumin on survival after an episode of HE, despite the higher survival observed in the albumin group, albumin failed to increase 90-day transplant-free survival in patients with cirrhosis combined with HE (91.9% vs. 80.5%, *p* = 0.3); competitive risk analysis of the data obtained observed 90-day cumulative mortality of 9% in the albumin group compared to 20% in the placebo group (*p* = 0.1) [39]. Another study has shown that albumin infusion does not prevent HE after transjugular intrahepatic portosystemic shunt (TIPS) [40]. In 2021, a randomized controlled trial study published in the New England Journal of Medicine, which included 777 hospitalized patients with decompensated cirrhosis combined with hypoproteinemia, showed no significant benefit of albumin infusion therapy compared to standard therapy in terms of the occurrence of infection, renal dysfunction, and mortality at 28 days, three months, and six months [41]. And the albumin group had more serious adverse events than the standard therapy group [41]. This study showed that patients with cirrhosis and HE who received albumin infusion had a higher risk score. This may be because patients need albumin infusion, which often means that the patient is in a state of hypoalbuminemia. Due to hypoproteinemia, the body’s immunity will decrease, and infections are prone to occur. Therefore, using albumin often indicates that the patient’s condition is serious and the prognosis is poor. In addition, infusion of more albumin is not completely safe. It is prone to serious adverse events, such as pulmonary edema or fluid overload [41, 42], which can even be life-threatening and affect the prognosis. In the future, more relevant clinical trials are needed to validate the efficacy of albumin infusion therapy and the doses used for cirrhosis combined with HE. As a drug for acid-related diseases, proton pump inhibitors (PPI) are widely used in liver cirrhosis patients, especially those with esophageal varices bleeding caused by portal hypertension. Several studies have shown that PPI therapy may increase the risk of HE in patients with cirrhosis [43–45], and the risk will increase with the dose of PPI [43]. PPI may inhibit gastric acid and promote intestinal flora overgrowth and translocation [46], thus increasing the incidence of HE. According to reports, PPI could increase the mortality of patients with liver cirrhosis and HE without active gastrointestinal bleeding [47]. However, a multicenter retrospective study found that for patients with cirrhosis, frequent treatment with PPI administration may increase the risk of HE incidence without worsening the prognosis of the patients [48]. In another single-center prospective study of 489 cirrhosis patients with or without acute-on-chronic liver failure, studied in subgroups with or without PPI therapy, it was found that PPI use did not increase mortality or the risk of HE in patients with cirrhosis [49]. This study found that using PPI has a protective effect on patients with liver cirrhosis combined with HE. Although the results of this study seem to contradict the results of some previous studies, it is important to consider that patients with liver cirrhosis complicated with HE are often in a period of decompensation and usually have other comorbidities, such as gastrointestinal bleeding caused by portal hypertension. In the short term, using PPI to deal with relevant indications promptly may improve the prognosis of patients. This study is retrospective and has limitations, and the specific indications for PPI use in each patient were not fully clarified. So, balancing PPI’s benefits and adverse effects still requires more prospective research to provide relevant proof.

## Limitation

This study has several limitations. First, this study was a single-center study with internal validation and a small sample size. Therefore, further large-scale prospective multi-center trials are needed to validate this prognostic nomogram. Secondly, the database could not fully capture the complete information of patients, and the missing partial data led to the reduction of sample size. Thirdly, some important indicators, such as blood ammonia and HE grade, were not included in this study because the data of these indicators were missing more than 20% or challenging to extract from the database.

## Conclusion

This study showed that older age, higher mean heart rate, lower MAP, lower mean temperature, higher SOFA score, higher RDW, and the use of albumin were risk factors for the prognosis of patients. The use of PPI was a protective factor. The C index, AUC value, calibration curve, IDI value, NRI value, and DCA curve are used to evaluate the performance of the nomogram, showing that the new nomogram has better performance than the SOFA model alone. The eight-factor nomogram has reasonable accuracy in predicting the 90-day survival rate of these patients. The results of this study may provide a reference for doctors to make clinical decisions on patients with HE.

## Data Availability

The datasets presented in this study can be found in MIMIC-III online repositories at https://physionet.org/content/mimiciii/1.4/, 10.13026/C2XW26.

## Abbreviations:

HE: Hepatic encephalopathy
C-index: Concordance index
AUC: Area under the curve
ROC: Receiver operating characteristic
NRI: Net reclassification improvement
IDI: Integrated discrimination improvement
DCA: Decision curve analysis
MIMIC-III: Medical Information Mart for Intensive Care III
MAP: Mean arterial pressure
SpO2: Blood oxygen saturation
ALT: Alanine aminotransferase
AST: Aspartate aminotransferase
ALP: Alkaline phosphatase
INR: International normalized ratio
PT: Prothrombin time
PTT: Partial thromboplastin time
PLT: Platelet
RDW: Red cell distribution width
WBC: White blood cell count
RBC: Red blood cell count
PPI: Proton pump inhibitor
PAD: Percutaneous abdominal drainage
SOFA: Sequential organ failure assessment
MELD: Model for end-stage liver disease
